# COVID-19 emergency cash transfer and pregnancy in Brazil

**DOI:** 10.4054/DemRes.2026.54.17

**Published:** 2026-03-17

**Authors:** Alexandre Gori Maia, Leticia Junqueira Marteleto, Luiz Gustavo Sereno, Sneha Kumar, Molly Dondero

**Affiliations:** 1 Universidade Estadual de Campinas, Campinas, Brazil.; 2 University of Pennsylvania, Philadelphia, PA, USA.; 3 University of Pennsylvania, Philadelphia, PA, USA.; 4 Northwestern University, Evanston, IL, USA.; 5 American University, Washington, DC, USA.

## Abstract

**BACKGROUND:**

Governmental financial assistance programs were widely adopted during COVID-19, but their impacts on women’s reproductive behavior remain unknown.

**OBJECTIVE:**

This paper evaluates how a key social policy instrument implemented during the COVID-19 pandemic – emergency aid – impacted the probability of pregnancy among young women. We also examine whether the use of effective contraceptive methods helps explain the heterogeneous effects observed across socioeconomic groups.

**METHODS:**

We draw on unique population-based longitudinal data from 2020 and 2021 tracking the reproductive lives of women aged 18–34 in Pernambuco, Brazil, to analyze how emergency aid targeted at vulnerable households impacted pregnancy. We generate estimates emphasizing causal relationships by controlling for the non-random allocation of aid using a quasi-experimental strategy, the inverse probability weighting regression adjustment.

**RESULTS:**

Results highlight a reduction in the probability of pregnancy among women receiving emergency aid during the pandemic. The negative impact was more pronounced among the least educated and youngest women and those who initially intended larger families. We also found positive effects of emergency aid on contraception use among these socioeconomic groups.

**CONCLUSIONS:**

One possible explanation is that the emergency financial assistance helped women weather disruptions in public health services and ensured more consistent use of effective contraception, thereby, at least temporarily, helping women avoid pregnancy during a period of heightened socioeconomic uncertainties. Because those initially intending larger families and those least educated are among the poorer groups of women, the effects of the aid are stronger for these groups and for younger women, who have a larger reproductive window to meet their fertility preferences, which affords them the possibility of pregnancy in less uncertain times.

**CONTRIBUTION:**

Combined, findings build on a broader literature on the impact of cash transfers on fertility outcomes by highlighting how these processes have played out at the height of a massive public health crisis.

## 1. Introduction

Governmental financial assistance was an important social policy in several countries to buffer against the economic fallout of the COVID-19 pandemic. Women faced a disproportionate share of the negative socioeconomic consequences of the pandemic, such as income loss and increased housework. This uneven burden has led demographers to examine whether the demographic consequences of the pandemic extend beyond mortality and morbidity to affect fertility intentions and behaviors (Berrington et al. 2021; Marteleto et al. 2023). Emerging evidence suggests the pandemic contributed to increases in intentions to avoid pregnancy (Kahn et al. 2021; Lin et al. 2021; Luppi, Arpino, and Rosina 2020; Marteleto et al. 2024; Novelli et al. 2021), that such intentions were higher early on but persisted throughout the pandemic (Lindberg et al. 2021), and that financial concerns are a primary factor underlying changes in intentions (Lindberg et al. 2021; Malicka, Mynarska, and Świderska 2021). However, to date, less research has examined how financial factors during the pandemic, such as governmental assistance, affected women’s reproductive behavior, particularly pregnancy.

Although previous studies have focused more broadly on the effects of cash transfers on fertility, such works have not considered the impacts of these policies during public health crises, which are characterized by high levels of generalized uncertainty and worry that can also affect fertility. Moreover, findings from previous studies about the association between cash transfer programs and fertility are mixed and nuanced, with variation in the direction and strength of the association and by population subgroup. For example, some studies have found positive short-term effects of cash transfer programs on fertility rates – that is, an increase in fertility rates and number of children (Cowan and Douds 2022; Soares and Lima 2021). Other studies have found a decline in the fertility rate (Olson, Clark, and Reynolds 2019), and still others find no significant effect of cash transfer programs on pregnancy (Dake et al. 2018). Moreover, these associations vary by characteristics such as age, socioeconomic status, and number of children in the household (Cowan and Douds 2022; Garganta et al. 2017; Olson, Clark, and Reynolds 2019). Other work (Marteleto et al. 2024) has linked emergency cash assistance to fertility intentions during the COVID-19 pandemic, finding that women who requested the governmental cash benefit were more likely to abandon initial intentions to have (more) children. Yet no research has examined whether cash assistance provided during the COVID-19 pandemic affected the probability of pregnancy and whether the impacts vary across important characteristics, such as socioeconomic status and age. Therefore additional research is needed to better understand whether and how financial assistance affects fertility in general, especially in times of generalized uncertainty, such as during novel infectious disease crises.

In this spirit, this study examines how governmental emergency aid during the COVID-19 pandemic is associated with the probability of subsequent pregnancy among women in Brazil. Using unique longitudinal data from a probabilistic sample of women of reproductive age in Pernambuco, Brazil, this study examines how receipt of emergency governmental cash assistance early in the pandemic is associated with pregnancy later in the pandemic and whether the association varies by sociodemographic characteristics. In doing so, the study makes a pioneering advance in showing the impact of emergency cash assistance on women’s reproductive behavior during a massive public health crisis.

## 2. The Brazilian context: Fertility and COVID-19

Before proceeding, we contextualize our study by providing a brief overview of fertility and COVID-19 patterns in Brazil. Brazil’s total fertility rate has been at below replacement levels since the mid-2000s (Coutinho and Golgher 2018), following declines associated with socioeconomic improvements (Potter, Schmertmann, and Cavenaghi 2002). The marked regional and socioeconomic differences in fertility, including adolescent fertility, have decreased over the last decades. Yet childbearing starts at generally young ages in Brazil compared to countries with similar below replacement fertility levels (Esteve and Florez-Paredes 2018). Fertility also varies significantly among sociodemographic groups. For example, women with lower levels of education and those living in the poorest regions (North and Northeast) are more likely to have more children than they desire (Alves de Carvalho, Wong, and Miranda-Ribeiro 2016).

It is also important to note that Pernambuco was the epicenter of the Zika epidemic in Brazil. In late 2015, reports indicated that the Zika virus in pregnancy could lead to developmental problems, ranging from severe birth defects like microcephaly to milder neurological issues that may appear later in life (Brasil et al. 2025). Studies have shown a remarkable decline in the overall fertility rate in Brazil during the Zika epidemic (Marteleto, Maia, and Rodrigues 2023). Proximity to the Zika epidemic may also have shaped women’s fertility intentions during the COVID-19 pandemic – for example, by influencing their perceptions of risks and worries about the new virus outbreak (Marteleto, Dondero, and Koepp 2023).

Fertility was at its lowest level when COVID-19 hit Brazil (Marteleto et al. 2022). The first case of COVID-19 in Brazil was confirmed in late February 2020, and the first case in Pernambuco was reported shortly afterward, in early March 2020. Initially, schools and cultural spaces were closed. By mid-March, malls, bars, restaurants, service establishments, civil construction, and intercity public transportation had also closed (State of Pernambuco 2020). By April, beaches and public parks had closed, and in early May, five municipalities in the metropolitan region of Recife were in full lockdown, with strict measures gradually easing in late June (Silva, Figueiredo Filho, and Fernandes 2020). However, public schools in Pernambuco remained closed until May 2021, 14 months after the initial closures (State of Pernambuco 2021).

To mitigate economic hardships caused by the COVID-19 crisis, the Brazilian government offered emergency aid in the form of cash assistance to those whose monthly per capita income was less than half the minimum wage (nearly 520 reais; about $100) and to individuals belonging to households with monthly per capita income below half the minimum wage (nearly 520 reais; about $100) or total monthly household income below three minimum wages (nearly 3,120 reais; about $600), and who were not formally employed or registered as individual micro-entrepreneurs. The initial amount was 600 reais per month (about $115) and could be granted to up to two eligible adults per household (Presidency of the Republic 2020a, 2020b). For single-parent families headed by women, the benefit was doubled to 1,200 reais per month (about $230). In September 2020, aid was extended for four more months, with payments of 300 reais per month (about $57) (Presidency of the Republic 2020c). In 2021, the aid was extended twice more, for a total of seven monthly transfers of 250 reais (about $45) (Presidency of the Republic 2021b, 2021a).

## 3. Theoretical background

A large body of literature has analyzed how policies benefiting families with children affect fertility. We situate our study within the literature focusing specifically on cash transfer programs. Conditional cash transfers (cash transfers that condition the benefits on the number of children, for example) usually have positive impacts on fertility. In Argentina, a poverty alleviation program that provided monthly cash transfers per child to disadvantaged households had a positive impact on the fertility rate of households with at least one child, although it had no significant effect on first births among eligible childless households (Garganta et al. 2017). Studies have also investigated the consequences of Brazil’s Bolsa Família program on fertility. Its benefits also depend on the number of children and adolescents in the family. While the program is thought to have reduced fertility among teens from beneficiary families (Olson, Clark, and Reynolds 2019), evidence on the overall impact on total fertility rates remains inconclusive (Rocha 2018; Simões and Soares 2012).

A more limited body of literature has analyzed the indirect or unintended effects of unconditional cash transfers on fertility, but no conclusive evidence of positive or negative effects has yet emerged. For example, a randomized controlled trial of an unconditional cash transfer targeted to families with a child in Zambia found no significant impact on fertility (Palermo et al. 2016). The authors suggest that the desire for more children may have offset the ability to achieve smaller ideal family sizes or increases in investments in existing children. In turn, a quasi-experimental study with an unconditional cash transfer program in Pakistan found a positive effect on fertility (Awaworyi Churchill et al. 2024), with the authors suggesting that the program might have increased the benefits of having a child by promoting parental leisure time and child health. Cowan and Douds (2022) also found that cash transfers increased fertility rates in Alaska, suggesting that the additional income might have alleviated economic constraints on reproductive health and autonomy, such as the inability to afford health care to support a healthy pregnancy.

Other work (Marteleto et al. 2024) has linked emergency cash assistance provided by the Brazilian government during the COVID-19 pandemic to fertility intentions, finding that women who *qualified* to request this governmental cash benefit were more likely to abandon initial intentions to have (more) children. However, women who *requested* this aid were more likely to switch from intending to forgo having (more) children to wanting to have (more) children. These findings align with research showing that flexibility in fertility intentions can reflect uncertainty about economic factors (Sennott and Yeatman 2012) and that food insecurity during the pandemic may be associated with childbearing intentions (Zimmerman et al. 2022).

Yet, to the best of our knowledge, no study has analyzed the impacts of cash transfers on fertility during public health crises. We integrate the general literature on cash transfer programs and fertility with literature on fertility rates and intentions during the COVID-19 pandemic to develop hypotheses about how cash transfers might be associated with fertility during health crises. We speculate that both material and psychological factors might underlie these associations.

The literature suggests that uncertainties caused by COVID-19 may have led to lower fertility rates, as people tend to avoid major life-changing events, such as having a child, during periods of uncertainty and economic instability (Tasneem et al. 2023). As a result of the pandemic, many women have chosen to postpone having more children or have decided not to have any (more) children (Marteleto et al. 2023). In localities where public family planning clinics were closed during the pandemic, women without resources may have also faced more challenges in accessing modern contraception (Charles et al. 2022). In this context, income from emergency aid may have helped women better plan their reproductive health by accessing contraception to delay or avoid pregnancy.

Cash transfers are also linked to social stigma and to women’s empowerment, which may also indirectly affect pregnancy. While some studies in Latin America indicate that beneficiaries of cash transfers are traditionally linked to low social stigma (Layton 2020; Morales Martinez and Gori Maia 2018), other studies suggest that such transfers may boost the self-esteem and agency of women by promoting economic independence, physical health, and psychosocial well-being (Hunter, Patel, and Sugiyama 2021; Sugiyama and Hunter 2020). Studies in Africa also suggest that cash transfers may empower women from poor households by enabling them to accumulate human capital through improved education, skills, and health, particularly reproductive and sexual health for adolescent women, thereby reducing early marriage and teen pregnancy (Austrian et al. 2024; Baird, McIntosh, and Özler 2019). Indeed, a study investigating the Brazilian Bolsa Família program found that it reduced fertility among teens from beneficiary families (Olson, Clark, and Reynolds 2019). At the same time, by boosting material resources and psychosocial well-being, such programs could also empower women to become pregnant should they desire.

This rationale guides us to test the first research hypothesis:

Hypothesis 1: Emergency aid during the COVID-19 pandemic is associated with lower probabilities of pregnancy. Resources from the cash transfer might enable women to plan reproductive health strategies, such as access to contraception, to avoid pregnancy during periods of crises and uncertainties.

Divergent results in prior studies examining the relationship between cash transfers and fertility may stem from heterogeneous effects shaped by surrounding socioeconomic conditions. This is because the effects of cash transfers on fertility may be moderated by the predominant reproductive constraints within each sociodemographic group and whether additional income can affect these constraints (Cowan and Douds 2022). For example, in Brazil, where nearly two-thirds of pregnancies are unplanned (Gallardo-Alvarado et al. 2025; Nilson et al. 2023), planned pregnancy is more common among socially advantaged groups, such as White women with higher levels of education (United Nations Population Fund 2018). Disruptions in public family planning services during the pandemic may have made it more challenging for women relying on these services to prevent unintended pregnancies (Coutinho et al. 2020). In this context, emergency aid may have enhanced women’s financial autonomy, enabling them to make reproductive decisions on their own terms. We can thus expect stronger effects of emergency aid among sociodemographic groups that are more likely to plan their pregnancies and depend on public services for reproductive health.

Cash transfers are also often associated with lower rates of early pregnancy in low- and middle-income countries (Kneale et al. 2023). Potential mediating factors include the fact that cash transfers can alleviate poverty, increase school attendance, and delay sexual debut (Handa et al. 2015). In Brazil, during the first year of the pandemic, the teenage pregnancy rate fell by 8.4%, which has been linked to factors such as social distancing and reduced exposure of adolescents to risky situations (Monteiro et al. 2023). However, the potential impact of emergency aid on this outcome is still unknown.

Given the potential heterogeneous effects of cash transfers, our second research hypothesis is:

Hypothesis 2: Emergency aid is expected to have stronger negative effects on the probability of pregnancy among socioeconomic groups that are more likely to rely on cash transfers to plan their reproductive lives.

Another important mechanism that may explain the links between cash transfers and fertility is contraceptive use. While existing evidence in low- and middle-income countries remains inconclusive (Khan et al. 2016; Kneale et al. 2023; Velasco, Chrysanthopoulou, and Galárraga 2020), effects may depend on socioeconomic settings. For example, a study in Peru indicated that cash transfers empowered women to prevent unwanted births by improving access to and utilization of modern birth control methods (Laszlo, Majid, and Renée 2024). In a resource-constrained setting caused by the pandemic, we may expect cash transfers to alleviate financial constraints, thereby increasing contraceptive use. In Brazil, the pill is the most common contraceptive method, supplied free of charge by the public health system, and its use is particularly prevalent among young women (Trindade et al. 2021). If emergency income helped women continue the use of long-term contraception during disruptions to public health services, we might also expect more negative effects on the probability of pregnancy among social groups that rely more heavily on the public provision of contraception, such as younger women. In this respect, our third hypothesis is:

Hypothesis 3: Women who are more likely to rely on long-term contraceptive methods freely available from the public health service, particularly young women, may have especially benefited from emergency aid during public health service disruptions by being able to maintain consistent use of effective contraception.

## 4. Material and methods

### 4.1 Data

We used panel survey data from the first two waves of the Decode Zika and COVID (DZC) Project, fielded in Pernambuco, a coastal state in northeastern Brazil known for its diverse socioeconomic and racial makeup. The first wave (W1) of data collection was conducted from May to October 2020, and the second wave (W2) was conducted from May to August 2021. In W1, we conducted phone interviews (averaging 25 minutes) with 3,889 women aged 18–34. These respondents were selected via random-digit dialing (RDD) from a sampling frame of more than 19 million numbers obtained from a government concession of random cell phone numbers. Using this sampling base, we can achieve a probabilistic baseline sample of 94% and 88% of women in the 18–34 age group who own a cell phone in the metropolitan region of Recife and in Pernambuco, respectively (IBGE 2021). The cooperation rate, defined as the proportion of eligible respondents who completed the interview among those successfully contacted, was 68.9%.

In W2, we recontacted our baseline respondents using a hierarchical, mixed-mode strategy that included phone, web, WhatsApp messages, and household visits (though interviews could not be conducted face to face due to COVID-19 protocols). We achieved a 66% follow-up rate, successfully reinterviewing 2,622 of our W1 respondents in W2. In W2 we inquired whether respondents had applied for and received COVID-19 emergency aid.

All analyses presented here are weighted using survey weights to account for selection probabilities and non-coverage error for W1, with additional non-response adjustments for W2.

### 4.2 Analytical sample

Because one criterion for eligibility was that the individual belonged to a household with a monthly family income of up to three times the minimum wage were eligible to receive the emergency aid, we restricted our analysis to 1,933 women (corresponding to 74% of the 2,622 women interviewed in both W1 and W2) with a family income up to 3,300 reais (nearly $630) to ensure comparability between the control and treatment groups. We do not have specific information on household per capita income, but according to data from the 2020 Brazilian National Household Survey (PNADC), individuals in Recife who meet the eligibility based on total household income (below three minimum wages) also meet the eligibility based on per capita income (below half of the minimum wage) (IBGE 2021). Further, we excluded 10 women (0.5% of the sample) who had undergone a hysterectomy or sterilization before W1 and 11 women (0.6% of the sample) with missing values for the independent variables (listwise deletion), resulting in a final analytical sample of 1,912 women. This aggregate sample size provides statistical power close to 100% to detect the effect of emergency aid on pregnancy at the 5% significance level, assuming the observed results from a linear probability model of pregnancy as a function of emergency aid and all explanatory variables described below. However, estimates disaggregated by category should be interpreted with caution, as the small number of pregnant women in our sample may compromise the precision of subgroup analyses (see Table A-1).

### 4.3 Variables

The dependent variable *Y* equals 1 if the woman had a pregnancy resulting in a live birth during the period of emergency aid (April 2020–April 2021). We used data from W1 and W2 to define this variable. First, we considered *Y* = 1 for pregnancies that commenced after the month of interview in W1 (May to October 2020) and persisted successfully until a live birth in 2021. We also considered *Y* = 1 for pregnancies during W2 that commenced from January 2021 onward and persisted successfully until the month of interview (between May and August 2021). The sample contains 71 women (3.9%) who were pregnant according to this definition.

The treatment variable *T* is equal to 1 if the woman or someone in her household received emergency aid in 2020 and is equal to 0 otherwise. Our sample includes 1,391 women (73%) who received emergency aid and 521 women (27%) who did not. Table A-1 also shows that 46 beneficiary women (3.3% of 1,391) and 25 non-beneficiary women (4.8% of 521) had pregnancies that resulted in live births.

We do not have detailed information on which household member received the benefit. However, because the emergency aid could be granted to up to two eligible adults per household and women accounted for more than half of all beneficiaries (Ministério da Cidadania 2021), most low-income women of reproductive age in our sample (who were probably not retired) likely received the transfer directly. The main cases in which an adult woman living in a beneficiary household might not have received the aid herself are when she was formally employed or when she lived with parents who were the registered beneficiaries. We account for these situations in our models using control variables such as formal employment, age, and a proxy for family status (explained below).

We also do not have detailed information on aid distribution, such as the total number of installments received, the amount of each benefit, or the difficulties respondents faced in accessing each installment. While this is mainly due to the confused implementation of COVID-19 emergency aid in Brazil, we recognize that this limits a more precise analysis of how aid amounts affect pregnancy outcomes. However, it is important to note that registration for emergency aid closed in July 2020, preventing further enrollments after that (Presidency of the Republic 2020a, 2020b). Individuals who received the first batch of payments (three installments) were automatically registered for the second batch (four installments). We can thus infer that if a woman reported receiving the aid, she received at least seven monthly installments, all disbursed by December 2020 (Presidency of the Republic 2020c).

The control variables (vector x) include a binary of 1 if the woman had never given birth to a live baby (nulliparous), a binary of 1 if in 2020 the woman reported wanting more children (wanted more), a binary of 1 if the woman lived with a partner in the same household (partner), a binary of 1 if the monthly household income was below 1,100 reais (poor), a binary of 1 if the woman was between 18 and 26 years old (young), a binary of 1 if the woman had some college education (college), a binary of 1 if the woman reported being Black (Black), a binary of 1 if the woman worked as a formal employee in the reference week (formal), and a binary of 1 if the woman worked as an informal employee in the reference week (informal). The reference category for the latter two binaries is a woman who did not work in the reference week.

### 4.4 Empirical strategy

We wanted to estimate the average treatment effect (ATE) – that is, the impact of emergency aid on the probability of pregnancy. First we fitted a probit model for the probability of being pregnant (*Y* = 1) as a function of *T* and covariates **x** using sampling weights (*w*_*i*_):

(1) Pr(Yi=1)=ϕ(δTi,x′𝛃i),

where *δ* represents the marginal effects of emergency aid on the probability of pregnancy and **β** is the vector of coefficients for the covariates. We fit the probit function ϕ using maximum likelihood estimators.

One limitation of this strategy is the potential for sample selection bias if the composition of the treatment and control groups differs on unobservable characteristics related to the probability of pregnancy. For example, access to information may affect both the probability of pregnancy and access to emergency aid.

We controlled for the lack of randomness among the beneficiaries of the emergency aid using a quasi-experimental strategy: the inverse probability weighting regression adjustment (IPWRA). The IPWRA is a two-stage method based on the propensity score, which is the probability of receiving the treatment conditional on covariates (Imbens and Wooldridge 2009). The IPWRA allows us to estimate the ATE by weighting individuals by the inverse probability of treatment, creating a synthetic sample where treatment assignment becomes independent of covariates (Austin and Stuart 2015). In the first stage, we use a probit model with sampling weights (*w*_*i*_) to fit the selection model for the probability of receiving emergency aid (*p*_*i*_):

(2) pi=Pr(Ti=1)=ϕ(x′𝛉i)

The propensity score *p*_*i*_ should balance the full set of pre-exposure confounders (Rosenbaum and Rubin 1983) – that is, variables that simultaneously affect the outcome (pregnancy) and the treatment (emergency aid). Achieving the correct balance requires either covariates related to the outcome or confounding covariates related to treatment and outcome (Austin and Stuart 2015; Kainz et al. 2017), which are represented by the variables in vector **x**.

In the second stage, we fit a probit model for the probability of being pregnant (the outcome model, Equation 1) using a combination of sampling weights and the inverse probability weights (IPWs: 1/*p*_*i*_ for *T*_*i*_ = 1 and 1/(1 – *p*_*i*_) for *T*_*i*_ = 0) of treatment or non-treatment estimated in the first stage (Ridgeway et al. 2015). One key advantage of IPWRA is that it is a doubly robust strategy, meaning the method obtains consistent estimates if either the treatment assignment (selection) or the outcome model is correctly specified (Imbens and Wooldridge 2009).

According to Imbens and Wooldridge (2009), the identification of the ATE using IPWRA estimators relies on two key assumptions: unconfoundedness and overlap. The unconfoundedness assumption states that, conditional on observed covariates, there are no unobserved confounders that are jointly associated with both the outcome and the treatment. In other words, we assume that the relevant confounders influencing both fertility and emergency aid are those included in vector **x**. The overlap (or common support) assumption asserts that for any relevant combination of covariates there should be both treated and untreated observations available for comparison. To assess the robustness of our results to this assumption, we compared IPWRA estimates with and without observations that may violate the common support assumption.

## 5. Results

### 5.1 The balance of the covariates

The estimates of the probit models for the probability of being a beneficiary of the emergency aid used to compute the IPW are presented in Table A-2. The main determinants of participation in the treatment (receiving emergency aid) are informal employment (positive association; *p*-value = 0.012) and a college education or more (negative association; *p*-value = 0.035). While only informal employees were eligible to receive the benefit, women with a college education or more represent the least vulnerable group among the sample of eligible women.

Table 1 shows descriptive statistics for the dependent and explanatory variables before and after IPW. Before IPW, the treatment group exhibited an overrepresentation of nulliparous, less educated, and employed women in the informal sector. For example, the proportion of informal employees among beneficiaries was 17 percentage points higher than among non-beneficiary women. After IPW, the treatment and control groups exhibit similar distributions of explanatory variables, indicating successful group balancing. While the average difference between the treatment and control groups (bias) reached 22 percentage points before IPW, it is equal to or lower than 3 percentage points for all variables after IPW. For example, before IPW, 64% of beneficiary women intended to have more children in 2020, compared to 50% of non-beneficiary women. After IPW, the proportion for both groups was 61%.

After IPW, the raw difference in the proportion of pregnant women between the treatment and control groups increased from −5.6 to −7.5 percentage points. When we compare beneficiary and non-beneficiary women with similar observable characteristics (after IPW), the percentage of women who became pregnant is 3.5% in the former group and 11% in the latter.

**Figure A-1:**
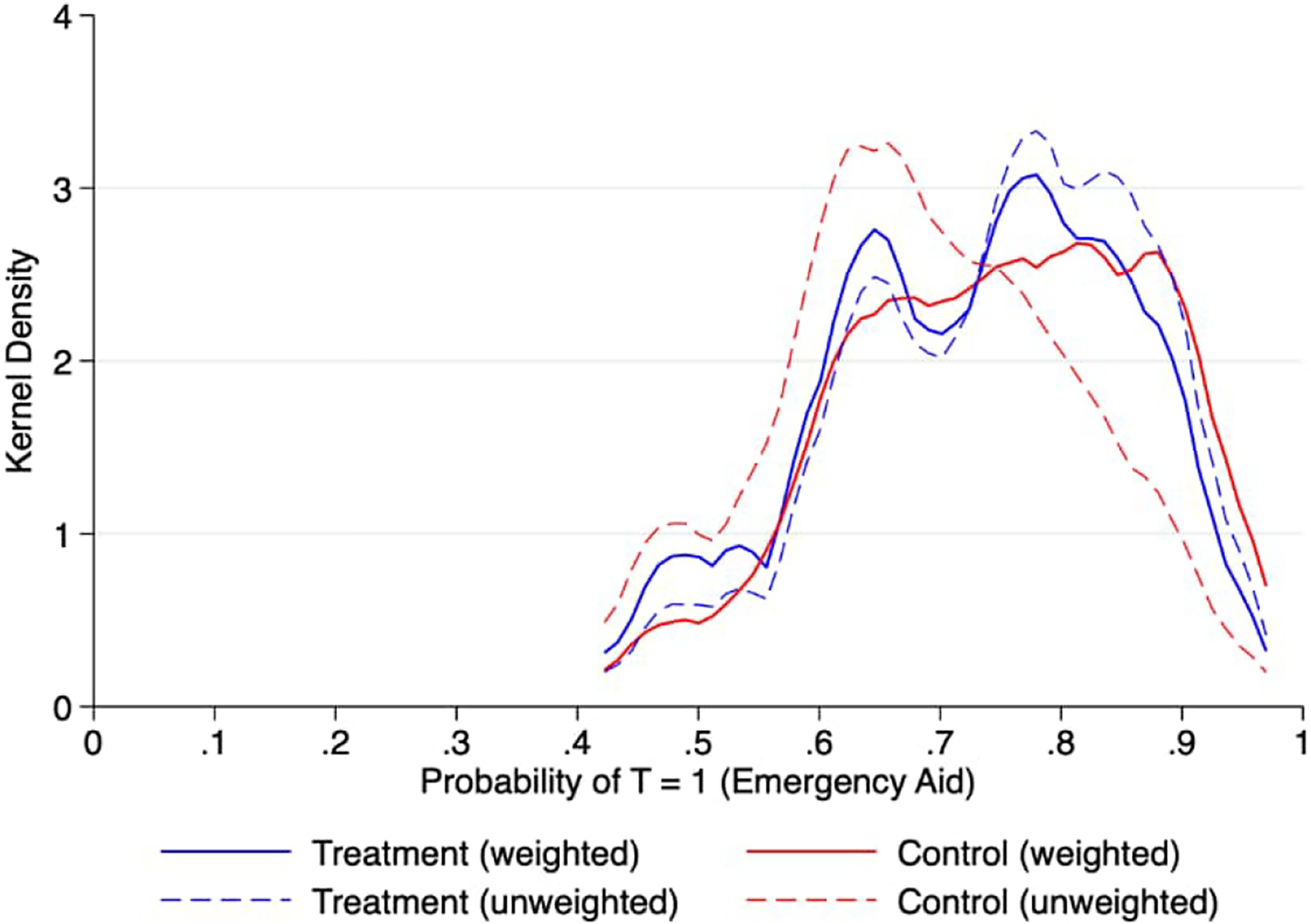
Kernel density of the probability of receiving emergency aid for the treatment group (blue) and the control group (red), before (dashed lines) and after (solid lines) weighting by the IPW (DZC Project, Pernambuco, Brazil) *Notes*: Estimates are based on a sample of 1,912 women aged 18–34. Control variables include binaries for nulliparous, wanted more children, poor, young, college or more, Black, formal employee, and informal employee.

An alternative strategy for assessing covariate balance and checking the common support assumption is to visualize the distribution of the estimated propensity scores (*p*) between the treatment and control groups. Figure A-1 shows the distributions for the treatment (blue) and control (red) groups before (dashed lines) and after (solid lines) weighting by the IPW. Prior to IPW, the control group exhibited an overrepresentation of women with a low probability of receiving emergency aid (*p*), indicating a potential imbalance in observable characteristics related to treatment assignment. After IPW, the treated and control groups have similar distributions, suggesting that IPW successfully balances observed covariates and produces more comparable groups.

**Figure A-2:**
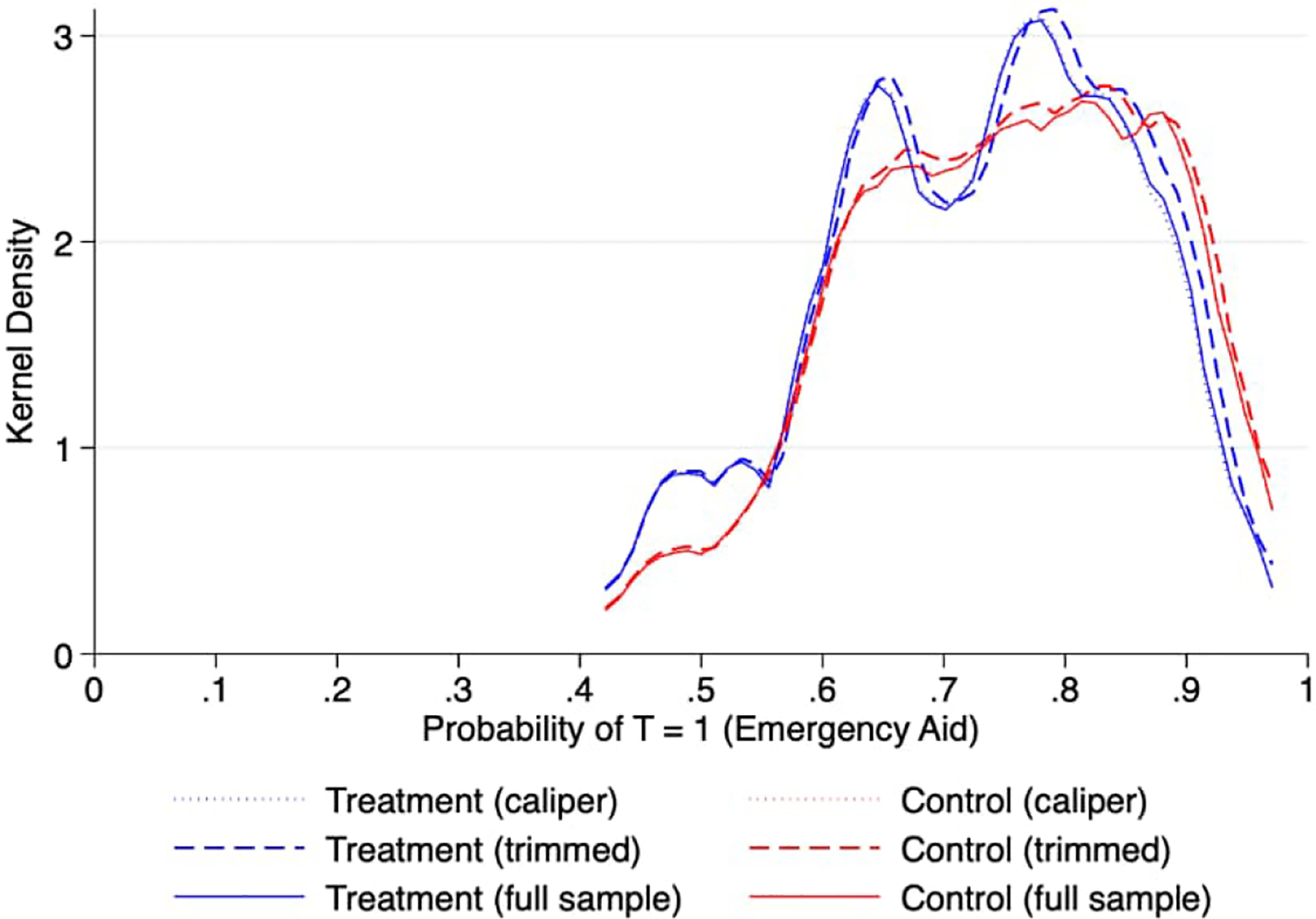
Kernel density of the probability of receiving emergency aid for the treatment group (blue) and the control group (red) after weighting by the IPW, for the full sample (solid lines), the trimmed sample (dashed lines), and the caliper-adjusted sample (dotted lines) (DZC Project, Pernambuco, Brazil) *Notes*: The full sample contains 1,912 women, the trimmed sample contains 1,881 women, and the caliper sample contains 1,902 women, aged 18–34. Control variables include binaries for nulliparous, wanted more children, poor, young, college or more, Black, formal employee, and informal employee.

We introduced two additional robustness checks to address potential violations of the common support assumption. First we restricted the sample by trimming observations with propensity scores above the 99th percentile (the trimmed sample). This strategy excluded 27 treated and 4 untreated women with *p* ≥ 0.93, who would otherwise have generated extreme weights (outliers). Second we restricted the sample to observations with at least one match within a 0.005 caliper. This strategy excluded ten treated observations that did not match a control counterpart within 0.005 points of their propensity score. Figure A-2 shows the distributions for the treatment (blue) and the control (red) groups, after applying IPW, for the full sample (solid lines), the trimmed sample (dashed lines), and the caliper-adjusted sample (dotted lines). While the trimmed sample slightly increased overlap, the three strategies exhibit broadly similar propensity score distributions for the treatment and control groups.

### 5.2 The effect of the emergency aid

Even after IPW, part of the difference in the probabilities of pregnancy may still be due to differences in the composition of the treatment and control groups, which are controlled for in Equation (1). Table 2 compares the estimates of the impacts of emergency aid on the probability of pregnancy with and without IPWRA. Estimates are robust across the two empirical strategies and indicate that emergency aid reduced the probability of pregnancy within a range of 2.4 (without IPWRA; *p*-value = 0.035) to 4.1 percentage points (with IPWRA; *p*-value = 0.034). Table A-3 shows similar estimates using IPWRA, restricted to the trimmed and caliper-adjusted subsamples, with ATT ranging from −3.9 (caliper-adjusted sample; *p*-value = 0.042) to −4.2 (trimmed sample; *p*-value = 0.033). The estimates of the four strategies also indicate that women who want more children and those living with partners in the same house are more likely to get pregnant.

**Figure 1:**
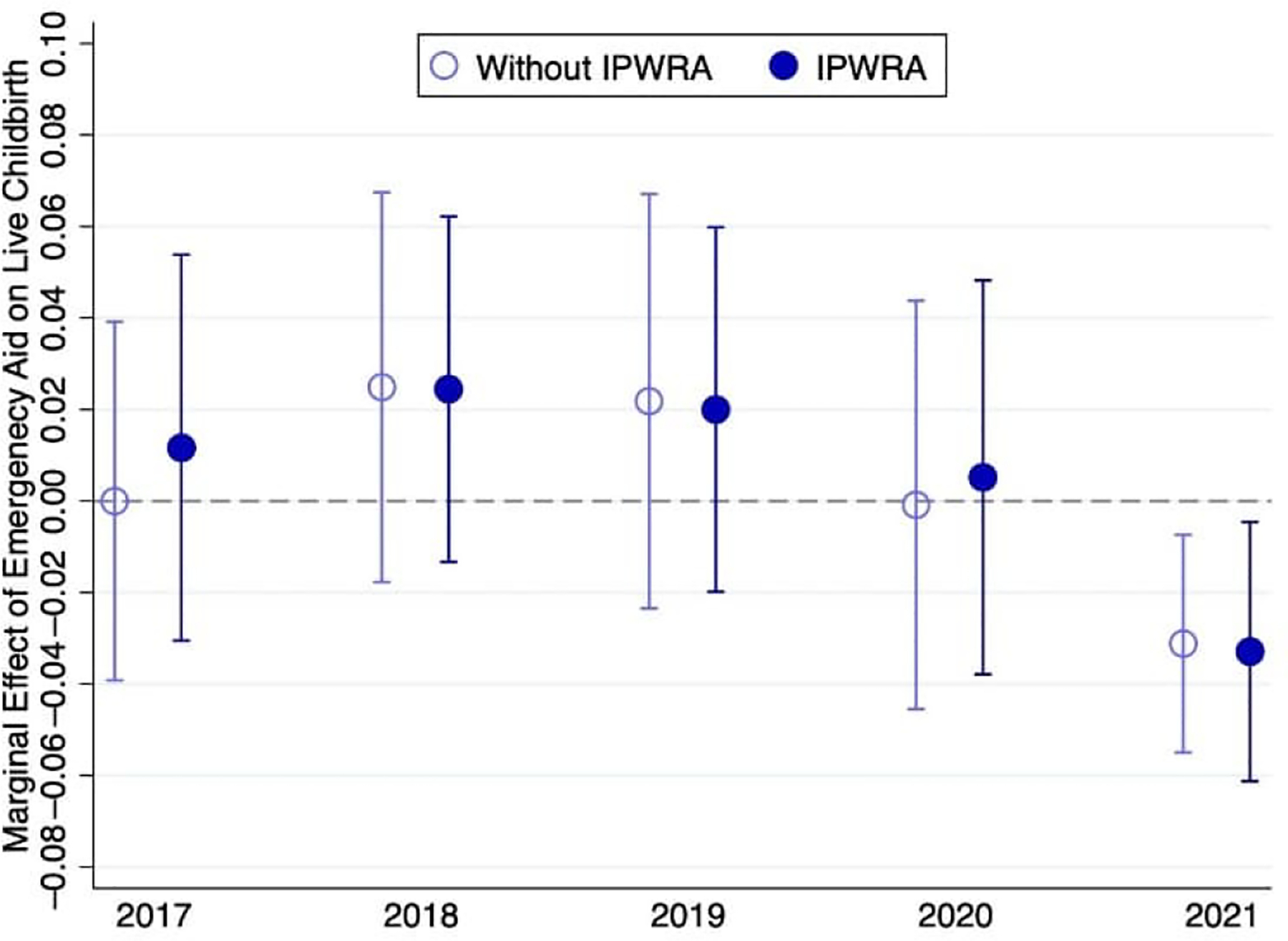
Estimates of marginal effects (and 95% confidence intervals) from probit models for the probability of live birth by year (DZC Project, Pernambuco, Brazil) *Notes:* Estimates are based on a sample of 1,912 women aged 18–34. Estimates without IPWRA use only sampling weights. Estimates with IPWRA use sampling weights and IPW. Control variables include binaries for nulliparous, wanted more children, poor, young, college education, Black, formal employee, and informal employee.

The similarity of the estimates with and without IPWRA suggests that selection on observable variables may not be a primary concern in our analysis. However, we may still have selection on unobservable variables. For example, the pregnancy rates of beneficiary and non-beneficiary women might have differed even before the former group began receiving the benefit, due to characteristics not captured by the covariates, such as social networks and cultural beliefs. We use the DZC childbirth history calendar to check whether the probability of live births for beneficiary women followed a trend similar to that of non-beneficiary women before emergency aid was implemented in 2020. Figure 1 shows the probability of having a live birth from 2017 (when all women in the sample were 13 years or older) to 2021 (the final year in the sample). We used the same empirical strategies as in Table 2, interacting the benefit of emergency aid with the year of the live birth. We found no compelling evidence of selection due to unobservable variables. Before the emergency aid was implemented (from 2017 to 2019), beneficiary and non-beneficiary women had an equal probability of childbirth. There was also no difference between the two groups in 2020, as many children born that year may have been conceived in 2019, before the implementation of emergency aid. In 2021 we observed a negative difference in the probability of live birth between beneficiary and non-beneficiary women.

### 5.3 Heterogenous effects

**Figure 2:**
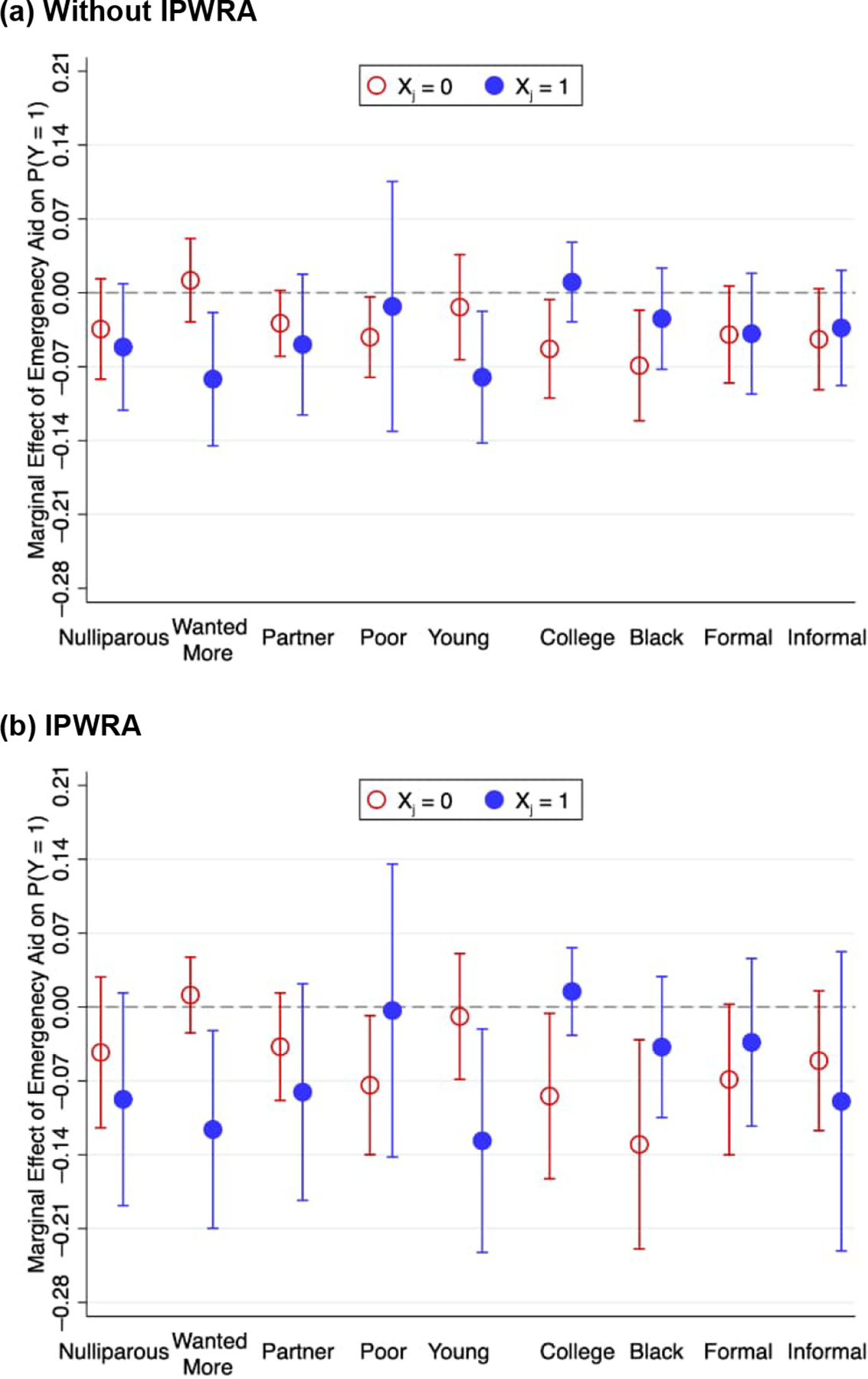
Estimates of the marginal effects (and 95% confidence intervals) from probit models for the probability of pregnancy resulting in live birth using interactions between emergency aid and control variables (X_j_) (DZC Project, Pernambuco, Brazil) *Notes:* Estimates are based on a sample of 1,912 women aged 18–34. Estimates without IPWRA (a) use only sampling weights. Estimates with IPWRA (b) use sampling weights and IPW. Control variables include binaries for nulliparous, wanted more children, poor, young, college, Black, formal employee, and informal employee.

We estimated separate models for the probability of pregnancy, interacting the benefit of emergency aid with each explanatory variable. The idea here is to identify potential mechanisms explaining the relationship between emergency aid and pregnancy. Figure 2 summarizes the results with marginal effect estimates and 95% confidence intervals for each category in each model (panel a, without IPWRA; panel b, with IPWRA). Table A-4 shows the significance tests for differences between these marginal effect estimates.

The main difference in the impact of emergency aid on pregnancy lies between women who wanted and women who did not want more children, ranging from 9 (without IPWRA; *p*-value = 0.012) to 13 percentage points (IPWRA with IV; *p*-value = 0.011). For example, the IPWRA estimates with IV indicate that emergency aid reduced the probability of getting pregnant by 11.6 percentage points among women who wanted more children but had little to no effect on women who did not want more children. We further explore this result in the next section.

We also found that the impact of emergency aid on pregnancy was more pronounced among those with no college education (*p*-value = 0.034) and younger women (*p*-value = 0.062). Emergency aid reduced by 8.4 percentage points (IPWRA estimates) the probability of pregnancy among women with no more than secondary education, while it had little to no effect on women with some college education. Similarly, while emergency aid reduced by 12.7 percentage points the probability of pregnancy among women between 18 and 26 years old, the impact was null among women 27 years or older.

### 5.4 Pregnancy not resulting in live birth and contraception

**Figure 3:**
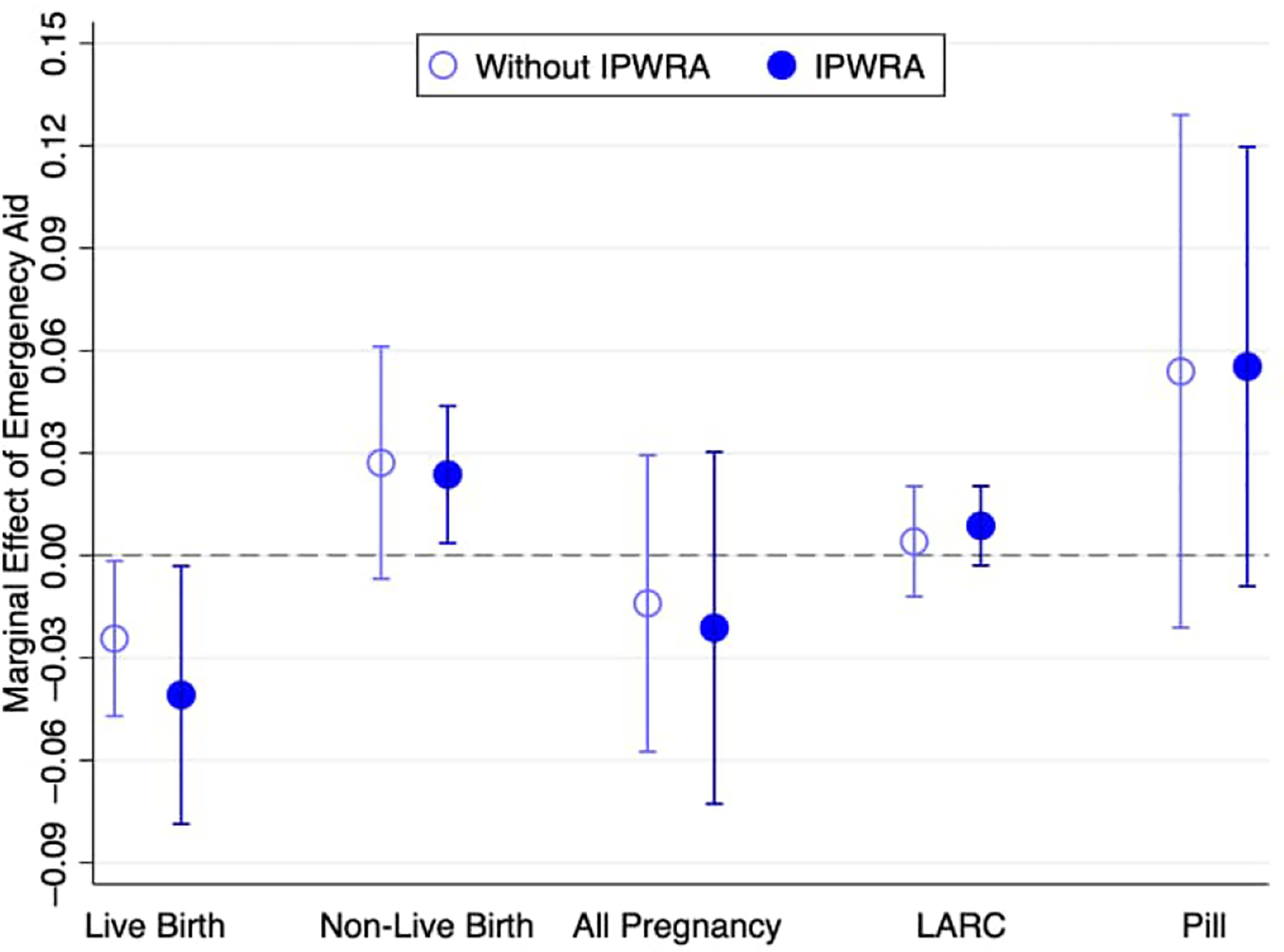
Estimates of the marginal effects (and 95% confidence intervals) from probit models for the probability of pregnancy resulting in live birth, pregnancy not resulting in live birth, total pregnancy (resulting in live birth or not), use of LARC, and use of the morning after pill (DZC Project, Pernambuco, Brazil) *Notes:* Estimates are based on a sample of 1,912 women aged 18–34. Estimates without IPWRA use only sampling weights. Estimates with IPWRA use sampling weights and IPW. Control variables include binaries for nulliparous, wanted more children, poor, young, college, Black, formal employee, and informal employee.

We tested potential mediators of the relationship between emergency aid and pregnancy, fitting models for four additional binary-dependent variables: pregnancy not resulting in live birth; all pregnancy (live and non-live birth); use of long-acting reversible contraception (LARC); and use of the morning after pill. The models use the same empirical strategies used in Table 2. Figure 3 shows the marginal effect estimates and 95% confidence intervals for each dependent variable. We also display the estimates for the live birth variable (from Table 2) for comparison.

The binary-dependent variable for pregnancy not resulting in live birth equals 1 for women who became pregnant between W1 and W2 but did not give birth to a living child. This group contains 39 women (2% of the sample) with non–live birth pregnancies due to intrauterine fetal demise, spontaneous abortion, or induced abortion. The IPWRA estimates are positive, suggesting that emergency aid increased the probability of pregnancy not resulting in live birth by 2.4 percentage points. As a result, the overall impact on all types of pregnancy (live and non-live births) was minimal to negligible.

The third binary-dependent variable, representing the use of LARC, assumes 1 for women who had undergone sterilization (or their partners had) and those using either a copper T IUD (intrauterine device) or a hormonal IUD. This group includes 35 women (2% of the sample) who used these contraception methods. The estimates suggest no association with emergency aid. One limitation of this variable is that it captures only current contraception use. The fourth binary-dependent variable assumes 1 for women who used the morning after pill. This group contains 274 women (14% of the sample) who used the morning after pill in this period. The point estimates are positive but imprecise, with confidence intervals spanning 0.

**Figure A-3:**
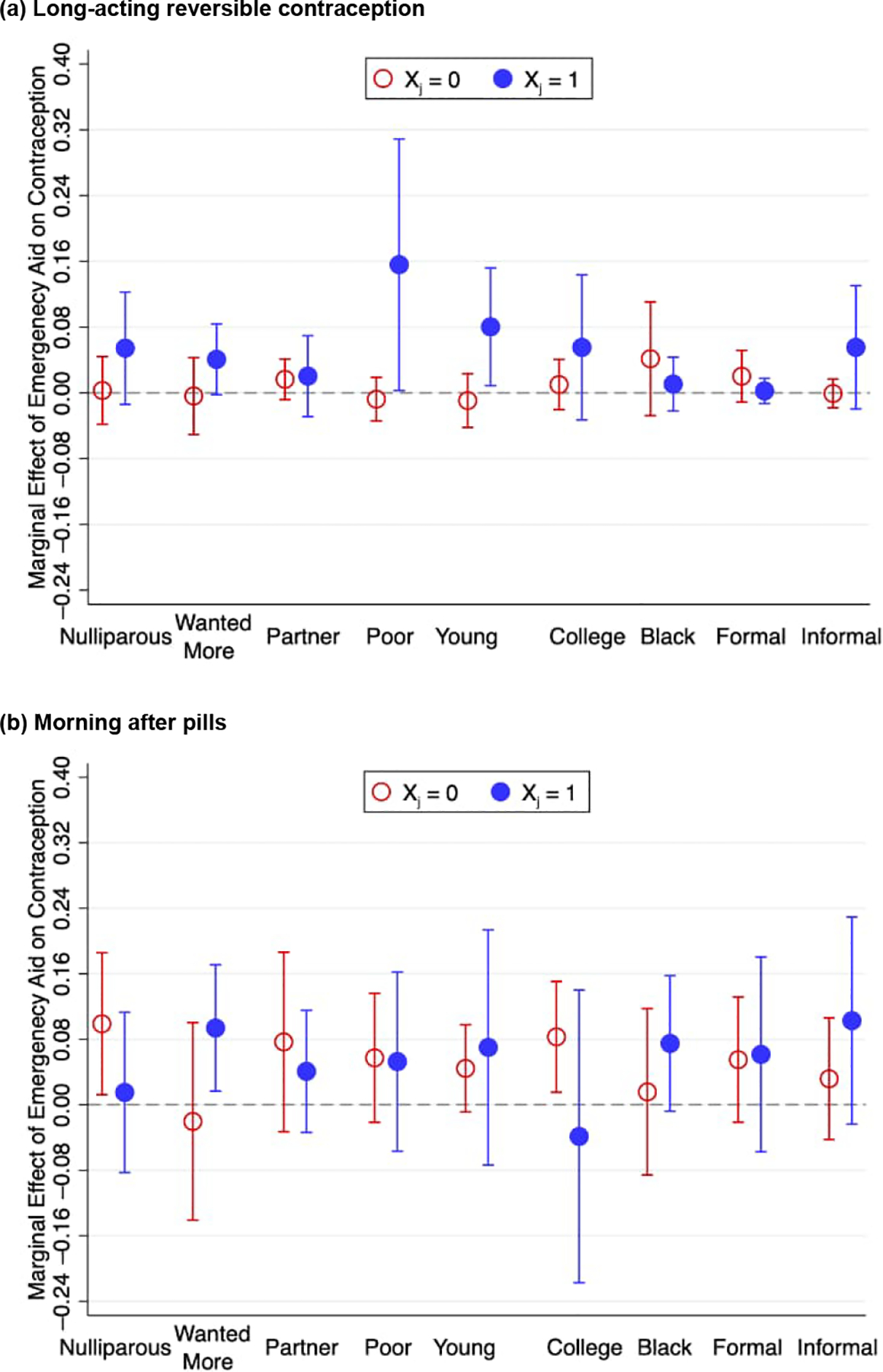
IPWRA estimates of the marginal effects (and 95% confidence intervals) from probit models for the probability of using (a) LARC and (b) morning after pills with interactions between emergency aid and control variables (X_j_) (DZC Project, Pernambuco, Brazil) *Notes*: Estimates are based on a sample of 1,912 women aged 18–34. All estimates use sampling weights and IPW. Control variables include binaries for nulliparous, wanted more children, poor, young, college, Black, formal employee, and informal employee.

Figure A-3 presents marginal effect estimates for models of the latter two dependent variables (use of LARC and use of morning after pill), where emergency aid interacts with each explanatory variable in separate models. We found that emergency aid increased the use of LARC among young women and increased the use of morning after pills among women who wanted more children and women without a college education. In other words, the use of LARC and morning after pills may help explain why emergency aid reduced successful pregnancies among younger women, less educated women, and women who wanted more children. While the marginal effect of emergency aid on the use of LARC is also positive for poor women, this estimate should be interpreted with caution, given the small sample size in this interaction and the consequent large standard error.

## 6. Discussion

The COVID-19 pandemic ushered in a period of high generalized uncertainty and fear alongside devastating health and socioeconomic consequences, with the most disadvantaged groups, such as women and individuals in low-income households, bearing the most severe burden of those consequences. Many governments around the world provided some form of emergency cash assistance to buffer the socioeconomic fallout of the pandemic, especially for the most vulnerable households. The unequal burden of the pandemic led to demographic repercussions that extended beyond mortality and health to fertility (United Nations Population Fund 2021). As demographers continue to chart patterns of fertility behaviors and intentions during and in the wake of the COVID-19 pandemic, our study examines an important and heretofore unexplored question: How was emergency cash assistance associated with pregnancy during the pandemic?

We used a unique population-based longitudinal dataset on young women in Pernambuco, Brazil, to address this question. Our findings revealed a negative association between receiving emergency aid during the COVID-19 pandemic and the probability of a subsequent pregnancy, with estimates indicating a reduction of 2.4 to 4.1 percentage points in the probability of pregnancy resulting in live birth. Our population-weighted estimates for the analytical sample indicate that 31,708 pregnancies resulted in live births among 686,887 women of reproductive age in Recife during the analysis period. Applying our estimated reduction in pregnancy probability (−2.4 to −4.1 percentage points) to the 514,099 women who received emergency aid, the program may have reduced the number of pregnancies by 12,338 to 21,078. These results underscore the discernible impact of financial support on women’s decisions regarding pregnancy, aligning with the notion that shifts in fertility timing preferences often occur in response to changes in life circumstances (Sennott and Yeatman 2012).

Additionally, our study indicates nuanced effects, suggesting that the impact was more pronounced among some sociodemographic groups of women – those who were younger, had no more than a secondary education, and expressed intentions to have larger families at baseline. This pattern resonates with the idea that the pandemic continues to disproportionately affect the sexual and reproductive health of individuals already facing systemic social and health inequities (Lindberg et al. 2021). These results contribute to the broader discussion of the interplay between social policies and reproductive decisions during public health crises. Findings also underscore the importance of considering women’s diverse socioeconomic and demographic characteristics when designing and implementing such assistance programs. While these results should be interpreted with caution due to the small sample of pregnant women, the findings align with the relationship we observed between emergency aid and the use of contraception.

One possible explanation for our findings is that government aid enabled women to use contraception more consistently and effectively, and to implement preferences to avoid childbearing, at least temporarily, until macrostructural conditions improved. The lack of monthly calendar data on contraceptive use (due to a shorter questionnaire required by a non-contact mode of data collection imposed by the pandemic) limits our ability to directly assess whether beneficiaries had consistent contraceptive use and whether they switched to a more effective method. However, the positive impact of the COVID-19 emergency cash transfer on the use of LARC and morning after pills among younger women and women with lower levels of education underscores this possible mechanism. Younger women may have been managing multiple uncertainties given their earlier life stage and at the same time had a longer reproductive window and the flexibility to delay having (more) children. Women with no college education likely experienced heightened economic insecurity during the pandemic and may have preferred to avoid children during this time to prevent strain on their limited resources. Aside from alleviating any income losses, emergency aid may have allowed young women to meet their reproductive desires and achieve more secure transitions to motherhood.

In alternate circumstances, one might expect the probability of pregnancy to increase following the receipt of aid, as aid could lower the direct cost as well as the opportunity cost of having children for (some) women. However, at the height of the COVID-19 pandemic, additional income from aid would not necessarily be sufficient to override the worry of getting pregnant and having children amid such heightened uncertainty. We contend that this worry was more acute for younger women and those in adverse socioeconomic conditions. Furthermore, because the pandemic in many ways compromised young children’s education and overall well-being, and emphasized the importance of parental investments, there may now be more motivation to have fewer children and to devote any additional income to improving a child’s quality of life.

Another mechanism that could explain our findings is welfare stigma – that is, the lack of self-respect and negative self-characterizations associated with welfare participation (Layton 2020; Morales Martinez and Gori Maia 2018). These negative feelings and subjective assessments linked with the receipt of aid could reduce the probability of pregnancy among beneficiaries if they wanted to limit further vulnerability and/or signal social responsibility via avoidance of pregnancy during COVID-19. Pregnancy avoidance was likely desirable social behavior during the pandemic in Brazil, given the heightened maternal mortality rates, compromised access to prenatal care, and unsafe childbirth conditions of this period (Guimarães 2023). Our findings of the negative impacts of emergency aid among women who wanted more children also reinforce the idea that the social stigma and income uncertainty brought by dependence on government assistance negatively influence expected pregnancies. Previous research has indicated that while economic stability enables couples to save money and plan childbirth more intentionally, the uncertainty women face regarding their future income levels – exacerbated by events like COVID-19 and dependence on governmental assistance – can lead to lower fertility rates, increased abortions, and the use of contraception (Ejrnæs and Jørgensen 2020).

An alternative hypothesis that is also linked to welfare stigma is aspiration. Women depending on cash transfers may decide to forgo pregnancies as a consequence of receiving aid because they are able to invest in human capital formation and avoid further economic hardships. Our finding of a positive association between emergency aid and pregnancies not resulting in live birth, which include those with deliberate termination, reinforces this explanation. This finding also aligns with previous literature indicating that economic insecurity is a primary reason why pregnant women may consider abortion (Roberts, Berglas, and Kimport 2020).

While our study underscores innovative findings using unique data, it has some limitations. The first set of limitations pertains to data. For instance, we do not have detailed data on aid access and disbursement (e.g., the total number of aid installments received or the difficulty eligible respondents had accessing each installment), a situation that precludes a more in-depth analysis of how aid impacted pregnancy outcomes. Similarly, data on pregnancies not resulting in live birth tend to be underestimated, as women frequently underreport both spontaneous and, above all, induced abortion in self-reported surveys (Jones and Kost 2007).

The second limitation is that our empirical strategy addresses selection based on observable characteristics – such as fertility attitudes and socioeconomic vulnerability – that are likely correlated with the sociodemographic controls used in our analysis. However, selection may also arise from unobservable factors – such as social norms and risk aversion – that could influence both reliance on assistance programs and fertility intentions. Despite this potential limitation, our robustness check found no evidence that selection based on unobservables is a major concern, as beneficiary and non-beneficiary women exhibited similar trends in pregnancy probability before the onset of COVID-19.

The third set of limitations pertains to external validity. Our results focus on women aged 18–34 living in the state of Pernambuco, excluding older women of childbearing age. While 18–34 is a wide age range, we may not necessarily find parallel results for women in older age groups, particularly if they desire (more) children – although it should be noted that most fertility in Brazil happens at ages younger than 34. Also, the state of Pernambuco tends to fare worse across various socioeconomic indicators than other Brazilian states, and the fertility implications of emergency aid for vulnerable families in Pernambuco may differ from those in other states. It is also worth noting that Pernambuco was an epicenter of the Zika epidemic of 2015–2017, which might have had implications for how women considered their fertility during the subsequent public health crisis (Marteleto, Dondero, and Koepp 2023). It is also possible that other types of emergency aid would have had different implications for fertility. COVID-19 emergency aid policies differed significantly across countries (e.g., targeted aid in Brazil vs. mostly universal aid under the CARES Act in the United States), and the implications of such aid for pregnancy outcomes might vary depending on the design and bundling of such policies across contexts.

Overall, our findings shed light on the connections between financial needs and fertility, with important nuances. The negative impact of COVID-19 emergency cash transfers on pregnancy was more pronounced among those who were less educated, were younger, and intended to have larger families, suggesting that such a policy might have provided these groups of women with the means to delay pregnancy. It is possible that the effect of the cash transfer will fade over time in the aftermath of the pandemic, as women readjust their fertility to improve macrostructural conditions. In this case, we would see a tempo but not a quantum effect on fertility. On the other hand, it is also possible that women who delayed pregnancy during the pandemic will end up forgoing pregnancy altogether, therefore impacting fertility rates in the long run. Further analysis encompassing more waves of panel data will allow us to address the long-term impact of emergency cash transfers implemented during the COVID-19 pandemic and their implications for different groups of women.

Given Brazil’s enduring below-replacement fertility levels and socioeconomic inequalities, the country offers an important case study with which to disentangle the implications of emergency aid policies for fertility. While Brazil’s fertility levels have been low since the last decade, there are sustained socioeconomic differences in fertility that point to women’s reproductive autonomy and contraceptive access. For example, our findings show that when provided with emergency aid, less educated women became more likely to avoid pregnancy during the pandemic. This suggests that emergency aid enabled women of low socioeconomic status to exercise their reproductive autonomy, thereby promoting social equity. Brazil is one of the most unequal countries in the world, but achieving higher fertility levels should not come at the expense of social equity or reproductive autonomy. Rather, the main policy implication is to create the conditions for all women to exercise their reproductive autonomy.

## Figures and Tables

**Table 1:** Descriptive statistics of dependent and independent variables, before and after IPW (DZC Project, Pernambuco, Brazil)

	Before IPW			After IPW		
	Control	Treatment	Bias	Control	Treatment	Bias
Pregnancy	0.088 (0.284)	0.032 (0.176)	−0.056	0.110 (0.313)	0.035 (0.183)	−0.075
Nulliparous	0.286 (0.452)	0.481 (0.500)	0.196	0.440 (0.497)	0.431 (0.495)	−0.009
Wanted more	0.502 (0.500)	0.638 (0.481)	0.136	0.606 (0.489)	0.605 (0.489)	−0.001
Live partner	0.606 (0.489)	0.514 (0.500)	−0.092	0.576 (0.495)	0.545 (0.498)	−0.031
Poor	0.256 (0.588)	0.310 (0.663)	0.053	0.264 (0.595)	0.293 (0.647)	0.029
Young	0.304 (0.460)	0.526 (0.499)	0.222	0.460 (0.499)	0.467 (0.499)	0.007
College	0.287 (0.453)	0.210 (0.407)	−0.077	0.239 (0.427)	0.233 (0.423)	−0.006
Black	0.676 (0.468)	0.693 (0.461)	0.017	0.702 (0.458)	0.688 (0.464)	−0.014
Formal employee	0.304 (0.460)	0.194 (0.396)	−0.110	0.230 (0.421)	0.225 (0.418)	−0.005
Informal employee	0.214 (0.410)	0.382 (0.486)	0.169	0.345 (0.476)	0.339 (0.474)	−0.006

*Notes*: Estimates are based on a sample of 1,912 women aged 18–34. Estimates without IPW use only sampling weights. Estimates with IPW use sampling weights and IPW. Bias is the difference between the estimates for the treatment group (beneficiaries) and the control group (non-beneficiaries).

**Table 2:** Estimates of the marginal effects (standard errors [SE] in parentheses) from probit models for the probability of being pregnant (DZC Project, Pernambuco, Brazil)

Variable	Without IPWRA		With IPWRA	
	Estimate (SE)	*p*-value	Estimate (SE)	*p*-value
Emergency aid	−0.024 (0.012)	0.035	−0.041 (0.019)	0.034
Nulliparous	−0.002 (0.011)	0.873	−0.001 (0.021)	0.961
Wanted more	0.032 (0.012)	0.008	0.059 (0.020)	0.003
Live partner	0.043 (0.012)	0.000	0.071 (0.025)	0.005
Poor	0.021 (0.014)	0.144	0.017 (0.024)	0.472
Young	0.003 (0.010)	0.759	0.017 (0.018)	0.342
College	−0.015 (0.012)	0.200	−0.040 (0.023)	0.092
Black	0.008 (0.010)	0.429	0.000 (0.018)	0.995
Formal employee	0.003 (0.011)	0.781	−0.003 (0.019)	0.874
Informal employee	−0.035 (0.013)	0.006	−0.032 (0.026)	0.230
Sample size	1,912		1,912	
Wald Chi-square	46.31	0.000	32.73	0.000
Pseudo-*R*^2^	0.187		0.207	

*Notes:* Estimates without IPWRA use only sampling weights. Estimates with IPWRA use sampling weights and IPW.

## Appendix

**Table A-1:** Sample size, percentage of women who became pregnant, and group differences across categories of explanatory variables (Xj) (DZC Project, Pernambuco, Brazil)

	Number of pregnancies		% of pregnancies		Difference in %		
	(X_j_ = 0)	(X_j_ = 1)	(X_j_ = 0) (A)	(X_j_ = 1) (B)	(B)-(A)	CI (95%)	
						LB	UB
Emergency aid	25	46	4.8	3.3	−1.5	−3.6	0.6
Nulliparous	43	28	4.3	3.1	−1.2	−2.9	0.5
Wanted more	16	55	1.9	5.0	3.1	1.5	4.7
Live partner	10	61	1.1	6.1	5.0	3.4	6.7
Poor	58	13	3.5	5.1	1.6	−1.2	4.5
Young	41	30	3.5	4.1	0.6	−1.2	2.3
College	48	23	3.8	3.5	−0.4	−2.1	1.4
Black	20	51	3.5	3.8	0.4	−1.4	2.2
Formal employee	56	15	4.0	3.0	−1.0	−2.8	0.8
Informal employee	55	16	4.3	2.5	−1.8	−3.5	−0.2

*Notes*: Estimates are based on a sample of 1,912 women aged 18–34. CI = confidence interval; LB = lower bound; UB = upper bound.

**Table A-2:** First-stage estimates of the marginal effects (standard errors [SE] in parentheses) from probit models for the probability of receiving emergency aid (DZC Project, Pernambuco, Brazil)

Variable	Estimate (SE)	*p*-value
Nulliparous	0.109 (0.059)	0.065
Wanted more	0.035 (0.050)	0.486
Live partner	−0.006 (0.046)	0.897
Poor	0.037 (0.061)	0.549
Young	0.087 (0.050)	0.080
College	−0.094 (0.045)	0.035
Black	−0.002 (0.045)	0.968
Formal employee	−0.038 (0.055)	0.491
Informal employee	0.122 (0.049)	0.012
Sample size	1,912	
Wald chi-square	37.15	0.000
Pseudo-*R*^2^	0.070	

*Notes*: Estimates are based on a sample of 1,912 women aged 18–34. Estimates use sampling weights.

**Table A-3:** Estimates of the marginal effects (standard errors [SE] in parentheses) from probit models for the probability of being pregnant, using the trimmed and the caliper-adjusted sample (DZC Project, Pernambuco, Brazil)

Variable	Trimmed		Caliper-adjusted	
	estimate (SE)	*p*-value	estimate (SE)	*p*-value
Emergency aid	−0.042 (0.020)	0.033	−0.039 (0.019)	0.042
Nulliparous	−0.001 (0.022)	0.968	−0.004 (0.022)	0.859
Wanted more	0.060 (0.020)	0.003	0.063 (0.020)	0.002
Live partner	0.072 (0.025)	0.004	0.072 (0.026)	0.005
Poor	0.018 (0.024)	0.459	0.022 (0.026)	0.400
Young	0.018 (0.019)	0.345	0.020 (0.019)	0.298
College	−0.040 (0.024)	0.094	−0.040 (0.024)	0.087
Black	0.000 (0.018)	0.986	−0.003 (0.018)	0.882
Formal employee	−0.003 (0.019)	0.878	−0.002 (0.020)	0.928
Informal employee	−0.032 (0.027)	0.246	−0.027 (0.027)	0.319
Sample size	1,881		1,902	
Wald chi-square	31.80	0.000	32.44	0.000
Pseudo-*R*^2^	0.206		0.209	

*Notes*: Estimates without IPWRA use only sampling weights. Estimates with IPWRA use sampling weights and IPW.

**Table A-4:** Differences between the marginal effect estimates (standard errors [SE] in parentheses) from probit models for the probability of pregnancy resulting in live birth using interactions between emergency aid and control variables (X_j_) (DZC Project, Pernambuco, Brazil)

Variable	Without IPWRA				IPWRA			
	(Xj = 0) (A)	(X_j_ = 1) (B)	Difference		(X_j_ = 0) (A)	(X_j_ = 1) (B)	Difference	
			(B)-(A)	*p*-value			(B)-(A)	*p*-value
Nulliparous	−0.034 (0.024)	−0.051 (0.031)	−0.017 (0.040)	0.672	−0.043 (0.036)	−0.087 (0.051)	−0.044 (0.064)	0.488
Wanted more	0.012 (0.020)	−0.082 (0.032)	−0.094 (0.037)	0.012	0.011 (0.018)	−0.116 (0.048)	−0.127 (0.050)	0.011
Live partner	−0.029 (0.016)	−0.049 (0.034)	−0.020 (0.037)	0.589	−0.038 (0.026)	−0.081 (0.052)	−0.043 (0.058)	0.460
Poor	−0.042 (0.020)	−0.013 (0.060)	0.029 (0.063)	0.645	−0.074 (0.034)	−0.003 (0.071)	0.071 (0.078)	0.364
Young	−0.014 (0.025)	−0.080 (0.032)	−0.066 (0.042)	0.115	−0.009 (0.030)	−0.127 (0.054)	−0.118 (0.063)	0.062
College or more	−0.053 (0.024)	0.010 (0.019)	0.063 (0.029)	0.031	−0.084 (0.040)	0.015 (0.021)	0.099 (0.047)	0.034
Black	−0.069 (0.027)	−0.024 (0.024)	0.044 (0.034)	0.193	−0.130 (0.051)	−0.038 (0.034)	0.092 (0.057)	0.106
Formal employee	−0.040 (0.023)	−0.039 (0.029)	0.001 (0.037)	0.982	−0.069 (0.036)	−0.034 (0.041)	0.035 (0.054)	0.513
Informal employee	−0.044 (0.024)	−0.033 (0.028)	0.011 (0.036)	0.765	−0.051 (0.034)	−0.089 (0.072)	−0.039 (0.081)	0.635

*Notes*: Estimates are based on a sample of 1,912 women aged 18–34. Estimates without IPWRA use only sampling weights. Estimates with IPWRA use sampling weights and IPW.
